# Physiological responses and transcriptome analysis of *Hemerocallis citrina* Baroni exposed to *Thrips palmi* feeding stress

**DOI:** 10.3389/fpls.2024.1361276

**Published:** 2024-05-14

**Authors:** Zhuonan Sun, Hui Shen, Zhongtao Chen, Ning Ma, Ye Yang, Hongxia Liu, Jie Li

**Affiliations:** ^1^ College of Plant Protection, Shanxi Agricultural University, Taigu, China; ^2^ College of Horticulture, Shanxi Agricultural University, Taigu, China

**Keywords:** *Hemerocallis citrina* Baroni, *Thrips palmi*, physiological responses, biochemical compounds, transcriptome, plant insect resistance

## Abstract

Thrips are serious pests of *Hemerocallis citrina* Baroni (daylily), affecting crop yield and quality. To defend against pests, daylily has evolved a set of sophisticated defense mechanisms. In the present study, induction of systemic resistance in *Hemerocallis citrina* ‘Datong Huanghua’ by *Thrips palmi* feeding was investigated at both biochemical and molecular levels. The soluble sugar content of daylily leaves was significantly lower than that in control check (CK) at all time points of feeding by *T. palmi*, whereas the amino acid and free fatty acid contents started to be significantly lower than those in CK after 7 days. Secondary metabolites such as tannins, flavonoids, and total phenols, which are harmful to the growth and reproduction of *T. palmi*, were increased significantly. The activities of defense enzymes such as peroxidase (POD), phenylalanine ammonia lyase (PAL), and polyphenol oxidase (PPO) were significantly increased, and the degree of damage to plants was reduced. The significant increase in protease inhibitor (PI) activity may lead to disrupted digestion and slower growth in *T. palmi*. Using RNA sequencing, 1,894 differentially expressed genes (DEGs) were identified between control and treatment groups at five timepoints. DEGs were mainly enriched in secondary metabolite synthesis, jasmonic acid (JA), salicylic acid (SA), and other defense hormone signal transduction pathways, defense enzyme synthesis, MAPK signaling, cell wall thickening, carbohydrate metabolism, photosynthesis, and other insect resistance pathways. Subsequently, 698 DEGs were predicted to be transcription factors, including bHLH and WRKY members related to biotic stress. WGCNA identified 18 hub genes in four key modules (Purple, Midnight blue, Blue, and Red) including MYB-like DNA-binding domain (TRINITY_DN2391_c0_g1, TRINITY_DN3285_c0_g1), zinc-finger of the FCS-type, C2-C2 (TRINITY_DN21050_c0_g2), and NPR1 (TRINITY_DN13045_c0_g1, TRINITY_DN855_c0_g2). The results indicate that biosynthesis of secondary metabolites, phenylalanine metabolism, PIs, and defense hormones pathways are involved in the induced resistance to *T. palmi* in daylily.

## 1 Introduction

*Hemerocallis citrina* Baroni (daylily) is a perennial herbaceous plant belonging to the Liliaceae family with edible flowers, medicinal properties, and ornamental functions. Daylilies are naturally distributed in East Asia, with the paramount diversity of species originating in Korea, Japan, and China, and have been cultivated for thousands of years (Matand et al., 2020; Misiukevičius et al., 2023). Thrips species such as *Frankliniella intonsa*, *Thrips palmi*, and *Frankliniella occidentalis* are common pests of daylily, causing plant damage. The life cycle of thrips includes five stages: egg, nymph, prepupa, pupa, and adult. Adults lay eggs in young plant tissues; 1st and 2nd instar nymphs are agile, and young plant tissues are their favorite feeding site; 3rd instar nymphs (prepupae) are no longer fed and pupated underground in the uppermost 3−5 cm soil layer; 4th instar nymphs (pupae) do not eat and pass the pupal stage in the soil layer (Cannon et al., 2007). The generational overlap of thrips is extensive, and it takes 15−20 days to complete the first generation, of which the egg duration is 5−7 days, and the adult duration is 7−10 days. The turn of spring and summer is the first peak of thrips infecting daylily (Dhall et al., 2021). The filing-sucking mouthparts of thrips damage the young leaves, tender stems, and flower buds of daylily. Thrips-infested plants exhibit slow growth, shortened internodes, and bent flower buds, which diminishes commercial value. When thrips were present in great numbers, the bud dropping rate of daylily was 31.65% higher than in controls, the actual bud dropping rate was as high as 99.62%, and it is the only insect pest that can lead to a completely failed harvest (Gao et al., 2021). In addition, owing to the small size of thrips, the high degree of concealment, the rapid reproduction, and the high incidence of drug resistance, it is difficult to achieve the desired control effect with a single insecticide (Steenbergen et al., 2018). Therefore, the safest and most effective strategy for thrips prevention and control is to utilize the insect resistance of the host plant. To this end, investigation of the physiological mechanisms of thrips resistance in daylily provides a basis for breeding insect-resistant plants.

Host plant damage by phytophagous insects alters plant nutrient content, production of toxic secondary metabolites, the activities of defense proteins and enzymes, and upregulates the expression of various defense-associated genes (Badenes-Pérez, 2022; Beran and Petschenka, 2022). The redistribution of certain nutrients and rapid synthesis of secondary metabolites in plants after pest infestation affects the feeding, growth, and development of pests, which in turn stimulates insect resistance (Erb and Reymond, 2019; Barbero and Maffei, 2023). Levels of soluble sugars, free amino acids, and soluble proteins in bean leaves decreased with increasing population density and feeding time of *F. occidentalis*, and were lower than those in control levels (Qian et al., 2018). Pest damage induces the accumulation of flavonoids in plants (Ramaroson et al., 2022); *Spodoptera litura* feeding stress has been shown to induce *Glycine max* to synthesize flavonoids (Du et al., 2019). Examples of herbivore-induced defense mechanisms are the accumulation of toxic chemicals such as benzoxazinoids (BXDs; chemical defense), glucosinolates, and alkaloids, which are classes of specialized metabolites that function as deterrents (Batyrshina et al., 2020). Chemical defense by BXDs in wheat showed a complex response at the leaf and phloem level that altered aphid feeding preference, and BXDs act as antifeedants to aphids (Singh et al., 2021a). In response to pest stress, defense-related enzyme systems in plants are activated. The main defense enzymes include peroxidase (POD), polyphenol oxidase (PPO), and phenylalanine ammonia lyase (PAL). Changes in the activities of these enzymes reflects the insect resistance of host plants to a certain extent (War et al., 2018). PAL is the rate-limiting enzyme in the phenylpropanoid metabolic pathway. Pest damage in plants initiates or upregulates phenylpropanoid metabolism, which increases PAL activity in damaged parts, resulting in a substantial accumulation of lignin in the cell wall and cell wall thickening, which prevents the spread of pests. Simultaneously, the increase in PAL activity increases the content of phytoalexins, which are toxic to phytophagous insects, and thereby prevent and control pests (Pant and Huang, 2022). Thrips feeding causes a significant accumulation of reactive oxygen species (ROS) in plants, leading to cell damage; plant PPO and POD remove excessive H_2_O_2_ and superoxide anions to maintain the dynamic balance of ROS, thus protecting plants against damage (Mouden and Leiss, 2021). Protease inhibitors (PIs) competitively and reversibly bind to intestinal proteases of herbivorous insects and allosterically bind to inhibitor-insensitive proteases to reduce protease hydrolysis activity, ultimately leading to slow growth and dysplasia in insects (Divekar et al., 2023). When herbivorous insects feed, plants are exposed to mechanical challenge in the form of tissue injury and chemical challenge caused by insect salivary secretions entering plant tissues. Subsequently, PI genes are induced at the wound site through transmission of signal molecules and amplification of the signal via a cascade, resulting in PI genes being expressed locally at the wound site and throughout the plant (Ferreira et al., 2023).

Transcriptome sequencing technology (RNA-Seq) has been frequently applied to study the interaction mechanisms between pests and hosts, and has become the main approach to explore gene expression. The transcriptome is a fundamental link between genomic and proteomic information associated with biological functions. Regulation of transcription level is the most studied and most important regulation strategy in organisms (Lowe et al., 2017; Paul et al., 2022). In plants exposed to insect feeding stress, defense signaling pathways are initiated, a series of physiological and biochemical reactions are induced, and expression of defense genes is activated (Whiteman and Tarnopol, 2021). The physiological and biochemical metabolism of plants is altered through signal transduction, transcriptional regulation, and gene expression, which improves the resistance of plants to pest stress (Du et al., 2020; Wani et al., 2022). *Sitobion avenae* feeding induces PAL gene expression in wheat (Van Eck et al., 2010). In cotton, *Helicoverpa armigera* feeding induces changes in the gene expression of lysyl oxidase (LOX), propylene oxide cyclase, and chalcone synthase, and activates plant defenses against pests at the molecular level (Chen et al., 2020). In response to insect feeding, plants initiate multiple hormone signaling pathways such as jasmonic acid (JA), salicylic acid (SA), and ethylene (ET), causing the accumulation of plant defense compounds, stimulating the expression of defense genes, and triggering the release of volatile substances, which further enhances the resistance of plants to herbivorous insects (Kersch-Becker and Thaler, 2019; Zhao et al., 2021). In maize, *Spodoptera litura* feeding significantly upregulateds defense-related genes, oxidative stress-related genes, transcriptional regulatory genes, protein synthesis genes, plant hormone-related genes, and genes related to primary and secondary metabolism (Singh et al., 2021b). In tobacco exposed to *Bemisia tabaci* stress, defense pathways such as ROS, PI synthesis, hormone metabolism, and WRKY were significantly upregulated, and plant resistance was enhanced (Wang et al., 2023b). Transcription factors play a key regulatory role in the battle between plants and herbivorous insects by regulating cellular activities via gene expression. Members of the WRKY, APETALA2/ethylene response factor (AP2/ERF), basic helix-loop-helix (bHLH), basic leucine zipper (bZIP), myeloblastosis-related (MYB), and NAC (no apical meristem/Arabidopsis transcription activation factor/cup-shaped cotyledon) families are involved in the regulation of plant disease and insect resistance networks (Tsuda and Somssich, 2015).

Herbivorous insect feeding initiates the inducible defense mechanism of plants, triggering a series of signal transduction and gene expression events, and the generation of defense substances. Inducible defense plays an more important role in the self-protection of plants (Maleck and Dietrich, 1999). At present, there are few reports on the physiological responses and omics differences of daylily in response to thrips feeding. In the present study, *H. citrina* ‘Datong Huanghua’ inoculated with *T. palmi* was used to determine the content of nutrients and secondary metabolites and defense enzyme activities in leaves to elucidate the physiological changes that induce pest defenses. Transcriptome analysis of thrips-infested leaves was performed with healthy leaves as controls. Differentially expressed genes (DEGs) were identified, and the main transcription factors and their expression patterns were analyzed. Key insect resistance genes were identified to elucidate the induced defense mechanism of daylily in response to *T. palmi*.

## 2 Materials and methods

### 2.1 Materials

Adults *T. palmi* individuals naturally occurring in daylily fields at the Horticultural Station of Shanxi Agricultural University were used as the source of test insects. The daylily variety used in the study was Datong Huanghua, which was planted at the Horticultural Station of Shanxi Agricultural University.

### 2.2 Seedling growth

The study was performed from March to June 2023 at the Horticultural Station of Shanxi Agricultural University. To prevent *T. palmi* and other pests, a 60-mesh insect-proof net was used to set up a net room, similar to a vegetable greenhouse, from west to east in the field to establish the experimental plot. After 45 days of seedling growth, the experiment began.

To establish the treatment group with induction of *T. palmi* (*T. palmi*-fed, abbreviated as TF), 1 day before the experiment, sufficient *T. palmi* were collected in the field and brought to the laboratory in a cage (118.7 × 100 × 100 cm) made of 60-mesh insect-proof net. *T. palmi* was starved for 12 h prior to the test, to ensure adequate feeding induction on plants. On the day of the experiment, *T. palmi* were transported to the net room in 50-mL centrifuge tubes, and *T. palmi* from one tube were released onto 3−4 plants such that there were ~90 individuals per plant; at least 15 plants were treated overall to ensure that three biological replicates could be sampled at each point. *T. palmi* concentrated on the upper–middle position of young leaves, and each plant had 6−7 such leaves. Datong Huanghua plants in this treatment group (TF) were individually covered with a 60-mesh insect-proof net to prevent *T. palmi* from escaping. In the control group (control check, abbreviated as CK), no insects were introduced, daylily plants were allowed to grow normally without any treatment in the net room, and each plant was individually covered with insect-proof net. Each treatment group included three biological replicates.

Plant leaves were collected at 1, 3, 5, 7, and 9 days after the introduction of *T. palmi* (named TF1−TF5), and leaves of CK group plants collected at the same time served as controls (named CK1−CK5). Three replicates were included at each stage, yielding five extractions with 30 samples in total, which were frozen in liquid nitrogen and stored at -80°C until future use.

### 2.3 Determination of plant nutrient content

The content of amino acids, free fatty acids, and soluble sugars in TF1−TF5 and CK1−CK5 was determined. Amino acids content was determined using an amino acids content determination kit (ninhydrin colorimetric method; 50T/48S) and a standard curve obtained using cysteine (Li et al., 2023a). Free fatty acids content was determined using a free fatty acids content determination kit (copper soap colorimetry; 50T/48S) and a standard curve obtained using palmitic acid (Wu and Shen, 2021). Soluble sugars content was determined using a plant soluble sugars content determination kit (anthrone colorimetry; 50T/48S) and a standard curve obtained using anhydrous glucose (Kwon et al., 2015). All kits were purchased from Beijing Solarbio Science & Technology Co., Ltd (Beijing, China). Data were summarized and processed using Microsoft Excel 2010 and statistically analyzed with SPSS software v20.0. The significance of the difference in nutrients between healthy daylily leaves and leaves fed on by *T. palmi* was tested by Tukey’s test (*p* < 0.05) and graph plotting using SigmaPlot 14.0 software. Data processing for secondary matter content and defense enzyme activities of daylily leaves before and after feeding by *T. palmi* was done in the same way as data processing for nutrient content determination.

### 2.4 Determination of plant secondary metabolites content

The content of tannins, flavonoids, and total phenols in TF1−TF5 and CK1−CK5 was determined. Tannins content was determined using a Tannins content determination kit (Folin-Ciocalteu colorimetric method; 50T/48S) and a standard curve obtained using tannic acid (Sharma et al., 2021). Flavonoids content was determined using a flavonoids content determination kit (AlCl3 colorimetric method; 50T/48S) and a standard curve obtained using rutin (Liu et al., 2022). Total phenols content was determined using a total phenols content determination kit (Folin-Ciocalteu colorimetric method; 50T/48S) and a standard curve obtained using catechol (Palacios et al., 2021). All kits were purchased from Beijing Solarbio Science & Technology Co., Ltd (Beijing, China).

### 2.5 Determination of plant defense enzyme activities

The activities of POD, PAL, PPO, and PI in TF1−TF5 and CK1−CK5 samples were determined. POD activity was determined using a POD test kit (guaiacol method; 50T/48S) (Li et al., 2019). PAL activity was determined using a PAL test kit (L-phenylalanine method; 50T/48S) (Shang et al., 2023). PPO activity was determined using a PPO test kit (pyrocatechol method; 50T/48S) (Wang et al., 2023a). All kits were purchased from Beijing Solarbio Science & Technology Co., Ltd, Beijing, China. PI activity was measured using a plant PI enzyme-linked immunosorbent assay kit (double-antibody sandwich method; 50T/48S) and a standard curve obtained using serine protease inhibin (Kumar et al., 2018).

### 2.6 Transcriptome sequencing and analysis

RNA was extracted from TF1−TF5 and CK1−CK5 samples using the TRIzol method (Wang et al., 2022). RNA integrity was assessed using 1% agarose gel electrophoresis, and the RIN value was determined using an Agilent 2100 bioanalyzer (Agilent Technologies Inc., Santa Clara, CA, USA). After RNA quality determination, the cDNA library was constructed and high-throughput sequencing was performed on an Illumina Hiseq platform (Shanghai Majorbio Bio-Pharm Technology Co., Ltd, Shanghai, China) with three biological replicates. Raw data obtained by sequencing were filtered to remove adapters and low-quality reads, and high-quality clean data was obtained. The base quality score (Q30) of clean data was determined. Trinity software was used to assemble the clean data obtained by sequencing to construct the UniGene library.

### 2.7 Identification and annotation of DEGs

The relative expression levels of each gene were determined using the Transcripts Per Million (TPM) standardization algorithm in FeatureCounts software (Vera Alvarez et al., 2019) and combined with gene transfer format (GTF) files describing genomic features. DESeq 2 (Qi et al., 2023) was used to compare the number of read counts between TF and CK groups, and differential expression analysis was performed on samples between the groups. Genes with *p*-adjust <0.05 and | log2FC | ≥1 after *p*-value correction were considered DEGs. DEGs were functionally annotated using the Gene Ontology (GO) database (http://www.geneontology.org/) and the Kyoto Encyclopedia of Genes and Genomes (KEGG) database (https://www.genome.jp/kegg/). Finally, transcription factors of DEGs were predicted using the Plant Transcription Factor Database (PlantTFDB; http://planttfdb.gao-lab.org/prediction.php/).

### 2.8 Identification and functional analysis of key modules for defense enzyme activities and secondary metabolites synthesis

We constructed a transcriptome expression matrix of leaves from the TF1-TF5 samples and screened for genes with TPM values <1. Furthermore, we used the WGCNA package (version 1.6.6) in R software (version 3.4.4) to construct a gene co-expression network. We selected β = 16 as the soft threshold for subsequent analysis and used the ‘blockwiseModules’ function to construct the gene network, with the following parameter settings: power = 6, TOMType = unsigned, maxBlockSize = 100 000, minModuleSize = 80, mergeCutHeight = 0.25, nThreads = 0; all other parameters were set to default values, and module feature genes for each module were calculated. We used the ‘exportNetworkToCytoscape’ function in the WGCNA package to export network relationships between genes in relevant modules, and Cytoscape software (version 3.7.1) was used to create graphs.

### 2.9 Quantitative real-time PCR analysis

Seven genes were selected randomly for qRT-PCR validation. RNA was reverse-transcribed using a PrimeScript RT Reagent Kit (Takara, Beijing, China). All procedures were conducted in accordance with the manufacturer’s instructions. The resulting cDNAs were quantified by TB Green Premix Ex Taq II (Takara). Each qRT-PCR experiment (15 μL) consisted of 7.5 μL of 2× SG Fast qPCR Master Mix, 0.6 μL of each primer (10 μM), 40 ng of cDNA template, and ddH_2_O to 15 μL. Thermal cycling involved an initial denaturation at 95°C for 3 min, followed by 40 cycles of denaturation at 95°C for 30s, annealing at 56°C for 30s, and extension at 72°C for 40s. Relative expression levels of genes were calculated using the 2^-△△CT^ method with the actin gene as an internal control, and the experiment was repeated at least three times. Primer sequences are listed in **Supplementary Table 1**.

## 3 Results

### 3.1 Effect of *T. palmi* feeding on plant nutrient content

**Figure 1:**
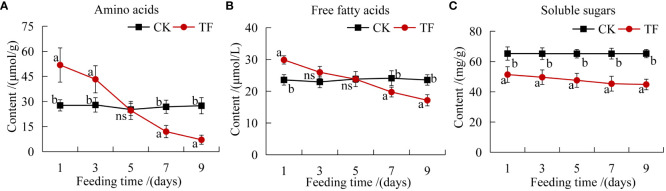
Determination of plant nutrients. **(A)** Amino acids content; **(B)** free fatty acids content; **(C)** soluble sugars content. Different letters indicate significant differences in nutrient composition between healthy leaves and leaves after feeding by *T. palmi* (*p <*0.05).

Primary metabolites such as amino acids, soluble sugars and free fatty acids play an important role in plant-induced defenses (Prado and Tjallingii, 2007). The amino acid and free fatty acid contents were higher than those in CK at 1 and 3 days, but significantly lower than those in CK at 7 days; they reached the lowest level at 9 days, 0.26 and 0.73 times the content in CK, respectively (**Figures 1A, B**). The soluble sugars content was significantly lower in the TF group than in the CK group at each timepoint and reached the lowest level at 9 day, 0.69 times that in CK (**Figure 1C**).

### 3.2 Effect of *T. palmi* feeding on plant secondary metabolites content

**Figure 2:**
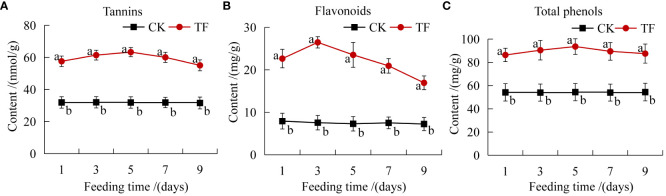
Determination of plant secondary metabolites. **(A)** Tannins content; **(B)** flavonoids content; **(C)** total phenols content. Different letters indicate significant differences in the content of secondary metabolites between healthy leaves and leaves after feeding by *T. palmi* (*p* < 0.05).

Insect feeding induces the accumulation of various toxic secondary metabolites such as phenols, alkaloids, and terpenoids in plants, and reduces the digestive capacity of insects and the amount of food and eggs, thereby directly or indirectly enhancing insect resistance (Mipeshwaree et al., 2023). Tannins, flavonoids, and total phenols in plants were increased significantly at each timepoint after *T. palmi* feeding induction. Flavonoids content reached a peak at 3 days, 3.5 times that in CK (**Figure 2B**). Tannins and total phenols content reached a peak at 5 days, 2 and 1.7 times that in CK, respectively (**Figures 2A, C**).

### 3.3 Effect of *T. palmi* feeding on the activities of plant defense enzymes

**Figure 3:**
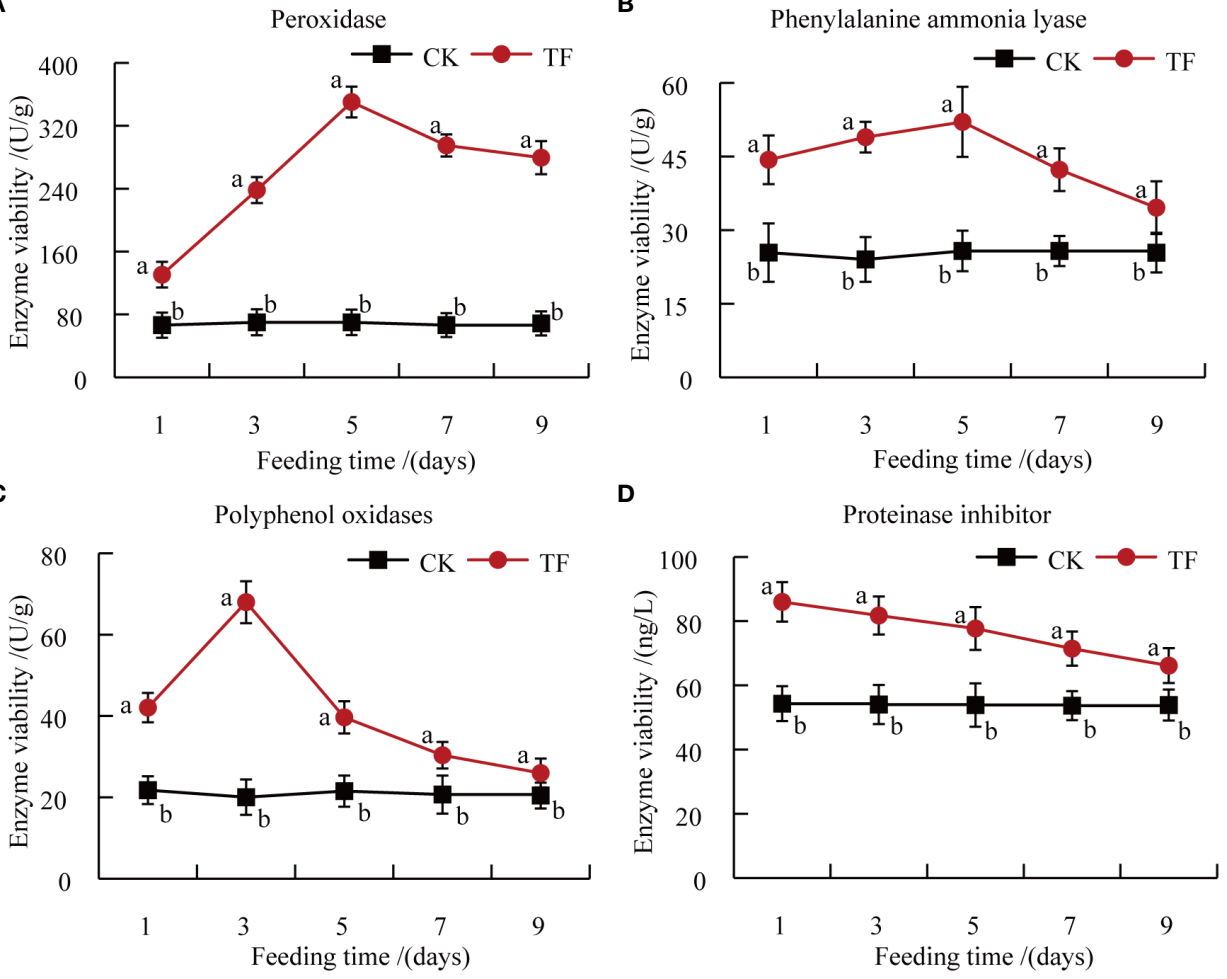
Determination of plant defense enzymes. **(A)** Peroxidase (POD) activity; **(B)** phenylalanine ammonia lyase (PAL) activity; **(C)** polyphenol oxidase (PPO) activity; **(D)** protease inhibitor (PI) activity. Different letters indicate significant differences in defense enzymes activities between healthy leaves and leaves after feeding by *T. palmi* (*p* < 0.05).

POD, PAL, PPO, and PI are defense enzymes of plants under biotic stress, and changes in these enzymes activities reflect the insect resistance of host plants to a certain extent (Uemura and Arimura, 2019). The activities of POD, PAL, PPO, and PI in leaves of Datong Huanghua induced by *T. palmi* feeding were significantly higher than in CK at each timepoint. POD, PAL, and PPO were all initially increased then decreased. POD and PAL activities reached a peak at 5 days, at 5 and 2 times those in CK, respectively (**Figures 3A, B**). PPO activity reached a peak at 3 days, 1.8 times that in CK (**Figure 3C**). PI activity reached a peak at 1 day, 1.6 times that in CK, and although it showed a downward trend, it was still 1.2-fold higher than in CK at 9 days (**Figure 3D**).

### 3.4 Quality assessment of transcriptome sequencing results

To study the changes in transcription levels of daylily under *T. palmi* stress, using Illumina 2× 150 bp paired-end sequencing, 141.58 Gb of clean data was obtained from 10 samples. Clean data from each sample reached >6.03 Gb, the percentage of Q30 bases was >94.18%. The percentage in brackets in the last column of **Table 1** is the comparison rate for clean reads; clean reads comparison efficiency ranged between 78.76% and 82.94%. The results showed that the quality of the sequencing output data was good, and the data could be used for further analysis.

**Table 1:** Transcriptome sequencing data statistics.

Sample	Raw reads/bp	Clean reads/bp	Q20 (%)	Q30 (%)	Mapped Reads/bp
CK1	48,597,229	43,722,323	96.95	94.35	17,805,077 (81.45%)
CK2	50,907,231	42,588,800	96.90	94.25	17,268,027 (81.10%)
CK3	46,682,755	41,929,344	96.94	94.36	17,363,380 (82.82%)
CK4	49,858,899	41,816,134	96.91	94.30	17,136,180 (81.96%)
CK5	52,020,018	42,876,465	96.87	94.18	17,551,857 (81.86%)
TF1	48,261,914	43,261,865	97.01	94.40	17,520,186 (81.00%)
TF2	50,083,781	43,001,949	96.92	94.29	16,936,456 (78.76%)
TF3	47,431,502	43,260,224	97.00	94.52	17,586,256 (81.31%)
TF4	44,050,892	42,486,957	97.11	94.72	17,617,214 (82.94%)
TF5	45,904,987	41,947,648	97.51	95.15	17,024,435 (81.18%)

### 3.5 DEGs in plants in response to *T. palmi* feeding

**Figure 4:**
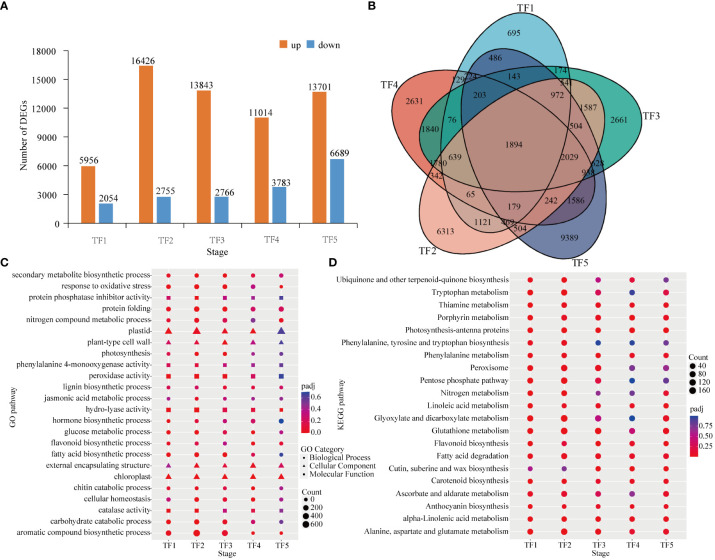
Differentially expressed genes (DEGs) in Datong Huanghua exposed to *Thrips palmi* feeding. **(A)** Number of DEGs at different stages; **(B)** venn diagram analysis of DEGs; **(C)** gene ontology (GO) enrichment analysis of DEGs; **(D)** Kyoto Encyclopedia of Genes and Genomes (KEGG) enrichment analysis of DEGs.

Transient expression of genes was investigated in the leaves of daylily in response to *T. palmi* feeding.The five timepoints after induction of *T. palm* feeding yielded 78,987 DEGs. The highest number of DEGs (20,390) was observed at the TF5 stage, including 13,701 upregulated and 6,689 downregulated genes. The TF1 stage had the lowest number of DEGs (8,010), including 5,956 upregulated and 2,054 downregulated genes. The number of upregulated genes was higher than that of downregulated genes at all stages (**Figure 4A**). Only 1,894 genes were differentially expressed at all stages (TF1−TF5; **Figure 4B**).

DEGs were subjected to GO functional annotation analysis to obtain their functions in response to *T. palmi* feeding. GO enrichment analysis showed that DEGs were more enriched in biological process (BP) subcategories, including secondary metabolite biosynthesis (GO:0044550), hormone biosynthesis (GO:0042446), fatty acid biosynthetic process (GO:0006633), jasmonic acid metabolism (GO:0009694), and lignin biosynthesis (GO:0009809). Among cellular component (CC) subcategories, plastids (GO:0009536) and plant-type cell wall (GO:0009505) were significantly enriched. Among molecular function (MF) subcategories, protein phosphatase inhibitor activity (GO:0004864), peroxidase activity (GO:0004601), and phenylalanine 4-monooxygenase activity (GO:0004505) were significantly enriched (**Figure 4C**).

Pathway analysis of DEGs was performed using the KEGG database to explore the metabolic processes and cell signaling pathways involved in genes associated with resistance to *T. palmi*. According to the KEGG enrichment analysis results for DEGs at the five stages, amino acid metabolic pathways such as α-linolenic acid metabolism, tryptophan metabolism, and phenylalanine metabolism, and plant insect resistance pathways including glutathione metabolism, flavonoid biosynthesis, anthocyanin biosynthesis, cutin, cork, and wax biosynthesis, and ascorbic acid and aldehyde acid metabolism, were significantly enriched after *T. palmi* feeding (**Figure 4D**).

### 3.6 Analysis of gene expression patterns related to *T. palmi* resistance

**Figure 5:**
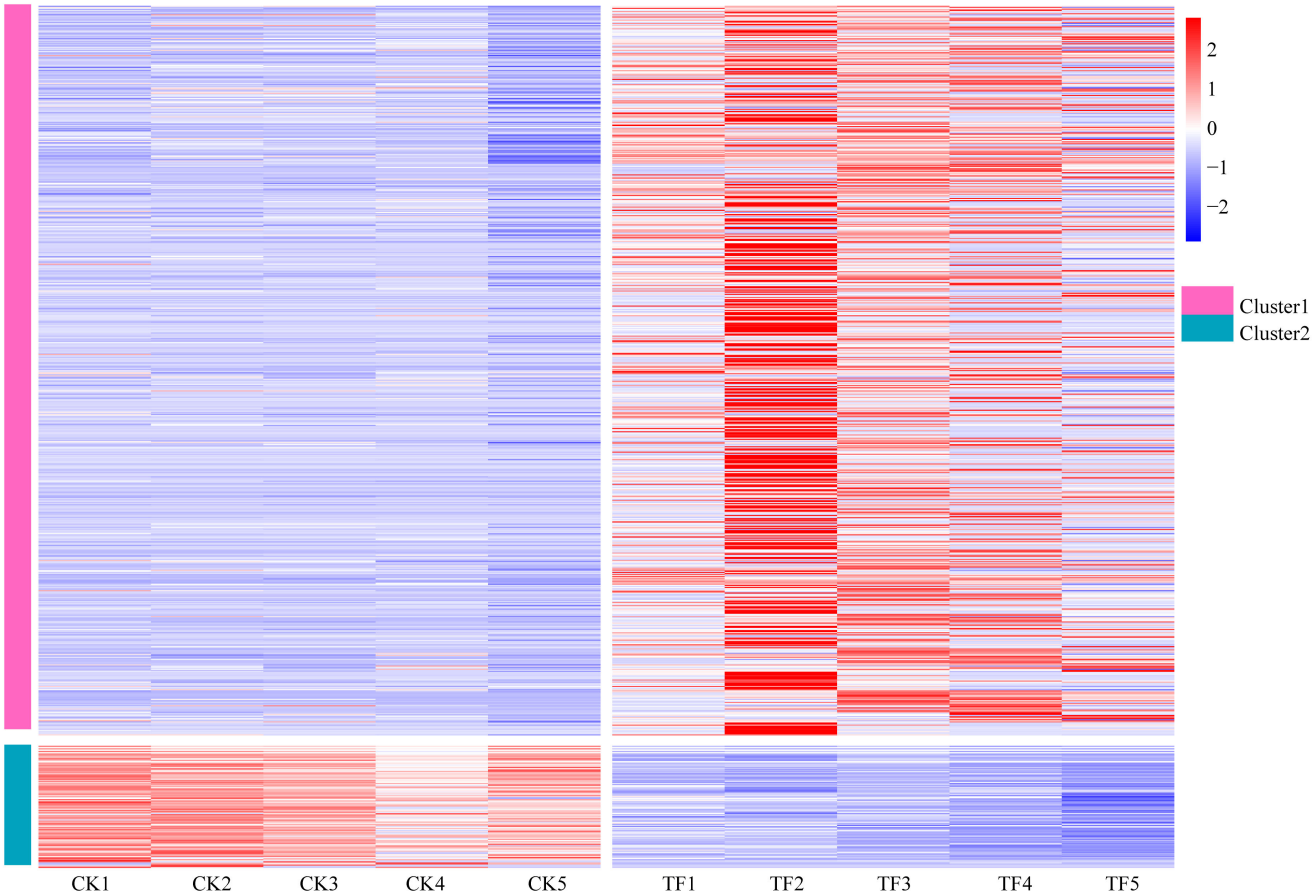
Analysis of gene expression patterns related to *T. palmi* resistance.

Based on the findings from DEGs, and GO enrichment and KEGG pathway analyses, 787 potential candidate genes related to *T. palmi* resistance were subjected to differential expression analysis (**Figure 5**). These genes could be divided into two expression patterns, among which Cluster 1 contains 679 genes. Its functions include the synthesis of secondary substances such as flavonoids, alkaloids and diterpenes, the synthesis of defense enzymes such as POD, PAL, PPO, PI, and catalase, the signal transduction of defense hormones such as JA and SA, MAPK signaling pathway-plant, wax synthesis, cell wall thickening, and others, which are mainly upregulated after feeding by *T. palmi*, and are more significant in the TF2 period. Cluster 2 contains 108 genes whose functions include amino acid metabolism, starch and sucrose metabolism, nitrogen metabolism, photosynthesis, carbohydrate metabolism, and others, which are mainly downregulated after feeding by *T. palmi*.

### 3.7 Prediction of transcription factors and their expression patterns

**Figure 6:**
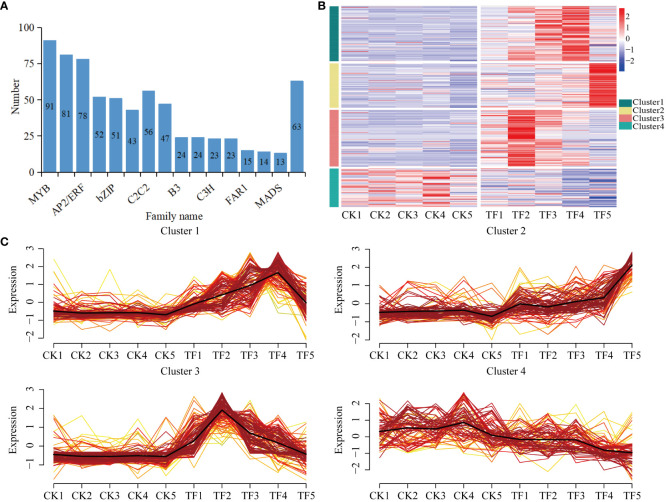
Expression of transcription factors. **(A)** Number of transcription factors; **(B)** transcription factors’ expression patterns; **(C)** expression pattern clustering results.

Transcription factors play a key role in the transcriptional regulatory network related to plant induced defenses. In order to explore the transcription factors related to *T. palmi* resistance in daylily, 698 transcription factors were identified from 78,987 DEGs, which clustered into 31 transcription factors families (**Figure 6A**, **Supplementary Table 2**). Approximately half of these genes are closely related to biological and non-biotic stress responses, including MYB, bHLH, AP2/ERF, WRKY, bZIP, and NAC. On the basis of their expression patterns, these genes were divided into four clusters (**Figures 6B, C**). The transcription factors in Cluster 1, Cluster 2, and Cluster 3 were upregulated after feeding by *T. palmi*. The transcription factors in Cluster 1 were mainly bHLH and WRKY, and were significantly upregulated at the TF4 stage. The transcription factors in Cluster 2, and Cluster 3 were mainly AP2/ERF and MYB, Cluster 2 was significantly upregulated at the TF5 stage, and Cluster 3 was significantly upregulated at the TF2 stage. The transcription factors in Cluster 4 were mainly bZIP and NAC, which were downregulated compared with CK. Cluster 3 and Cluster 1 included the highest numbers, with 107 and 104 upregulated transcription factors, respectively, indicating that they play an important role in the resistance of daylily to *T. palmi*.

### 3.8 Co-expression network identification and key module analysis

**Figure 7:**
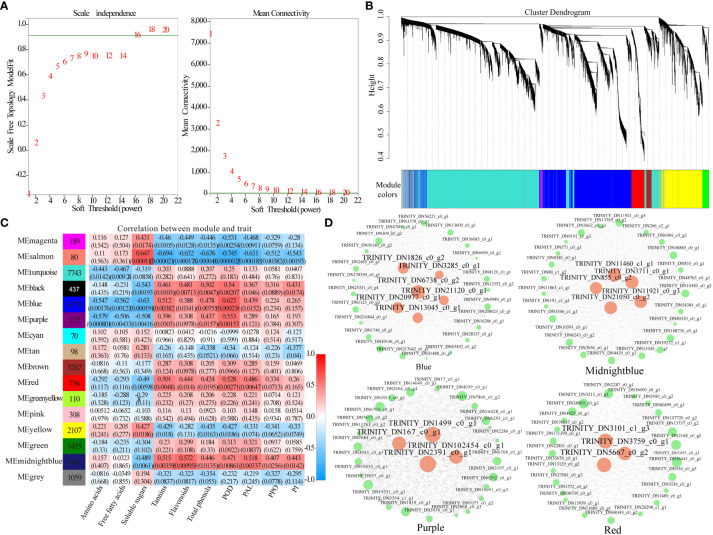
Weighted gene co-expression network analysis (WGCNA) of plant defense-related genes. **(A)** Scale-free network model index under different soft thresholds; **(B)** gene clustering tree based on the topological dissimilarity matrix; **(C)** heatmap of correlations between modules and traits; **(D)** gene co-expression network in the plant defense-related gene module; hub genes are colored pink.

WGCNA can be used to identify co-expressed gene modules, explore biological correlations between modules and target traits, and mine core genes in the module network. WGCNA was applied to the transcriptomic data to explore the relationships between genes related to the content of amino acids, free fatty acids, soluble sugars, tannins, flavonoids, and total phenols, and the activities of POD, PAL, PPO, and PI in daylily. The soft threshold β = 16 was determined by calculation (**Figure 7A**), and 24,665 genes were used to construct a co-expression network with 16 co-expression modules, among which the Turquoise module was the largest with 7,743 genes, whereas the Midnight blue module was the smallest with only 44 genes (**Figures 7B, C**). The Midnight blue module contained genes strongly linked to flavonoids content, PAL activity, tannins content, PI activity, and PPO activity, with correlation values of 0.572, 0.518, 0.515, 0.443, and 0.407, respectively. The Salmon module included genes strongly linked to soluble sugars content, with a correlation value of 0.647. The Black module contained genes strongly linked to total phenols content, with a correlation value of 0.502. The Blue module included genes strongly linked to POD, with a correlation value of 0.623. The Yellow module contained genes weakly linked to amino acids content and free fatty acids content, with correlation values of 0.221 and 0.205, respectively (**Figure 7C**). Four key modules (Purple, Midnight blue, Blue, and Red) highly correlated with the 10 phenotypes (amino acids, free fatty acids, soluble sugars, tannins, flavonoids, total phenols, POD, PAL, PPO, and PI) were selected, and key genes in the regulatory network were visualized using Cytoscape 2.0 with weights >0.4 (**Figure 7D**). A total of 18 network hub genes were identified as key genes and were annotated using *Arabidopsis* and *Asparagus* databases. Examples include natural resistance-associated macrophage protein, cytochrome P450, secondary metabolites biosynthesis, jasmonic/salicylic acid mediated signaling pathway, protein serine/threonine kinase activity, dienelactone biosynthetic, brassinosteroid biosynthetic, endonuclease/exonuclease/phosphatase family, glutamyl endopeptidase, haloacid dehalogenase-like hydrolase, and oxylipin biosynthetic process (**Table 2**). TRINITY_DN6738_c0_g2 plays a major regulatory role in the secondary material synthesis pathway, which influences pest feeding; TRINITY_DN21120_c0_g1 promotes the synthesis of PIs and hinders the digestive function of pests; TRINITY_DN167_c0_g1 regulates nutrient redistribution by plant amino acid metabolism to reduce the nutrients available to pests while ensuring normal plant growth; TRINITY_DN855_c0_g2 regulates defense hormone signaling, such as JA and SA, to activate plant systemic resistance. In addition, three transcription factors were annotated, namely MYB-like DNA-binding domain (TRINITY_DN2391_c0_g1, TRINITY_DN3285_c0_g1), zinc-finger of the FCS-type, C2-C2 (TRINITY_DN21050_c0_g2), and regulatory protein NPR1 OS (TRINITY_DN13045_c0_g1, TRINITY_DN855_c0_g2).

**Table 2:** Hub genes and predicted functions.

Gene ID	Homologous species/gene	Gene function
TRINITY_DN3285_c0_g1	*Telopea speciosissima* XP_043691361.1	MYB-like DNA-binding domain
TRINITY_DN1826_c0_g2	*Dioscorea alata* KAH7671728.1	Oxidative phosphorylation; Haloacid dehalogenase-like hydrolase
TRINITY_DN6738_c0_g2	*Asparagus officinalis* XP_020261199.1	Cytochrome P450; Secondary metabolites biosynthesis; Brassinosteroid biosynthetic
TRINITY_DN21120_c0_g1	*Asparagus officinalis* XP_020272828.1	Protein serine/threonine kinase activity
TRINITY_DN20977_c0_g1	*Elaeis guineensis* XP_010939670.1	Phosphate-induced protein
TRINITY_DN13045_c0_g1	*Dendrobium catenatum* PKU61926.1	NPR1-interacting
TRINITY_DN11460_c1_g1	*Ananas comosus* XP_020082461.1	AWPM-19-like membrane family protein
TRINITY_DN3711_c0_g1	*Asparagus officinalis* XP_020244100.1	Natural resistance-associated macrophage protein
TRINITY_DN855_c0_g2	*Castanea mollissima* KAF3962412.1	Jasmonic/Salicylic acid-mediated signaling pathway; Regulatory protein NPR1
TRINITY_DN11921_c0_g6	*Dioscorea alata* KAH7666769.1	Endonuclease/Exonuclease/phosphatase family
TRINITY_DN21050_c0_g2	*Asparagus officinalis* XP_020249010.1	Zinc-finger of the FCS-type, C2-C2
TRINITY_DN1499_c0_g1	*Asparagus officinalis* XP_020268739.1	Transcript variant X3, mRNA
TRINITY_DN167_c0_g1	*Asparagus officinalis* ONK67613.1	Glutamyl endopeptidase; Amino acid metabolism; Dienelactone biosynthetic
TRINITY_DN102454_c0_g1	*Dendrobium nobile* KAI0496278.1	Cullin-3A-like
TRINITY_DN2391_c0_g1	*Asparagus officinalis* XP_020250343.1	MYB-like DNA-binding domain
TRINITY_DN3101_c1_g3	*Capsicum annuum* XP_016565577.1	Cysteine-rich receptor-like protein kinase 31;oxylipin biosynthetic process
TRINITY_DN3759_c0_g1	*Dendrobium chrysotoxum* KAH0445920.1	Cytosolic large ribosomal subunit
TRINITY_DN5667_c0_g2	*Quercus suber* XP_023907021.1	EXF-150 Actin

### 3.9 Verification using quantitative real-time PCR

**Figure 8:**
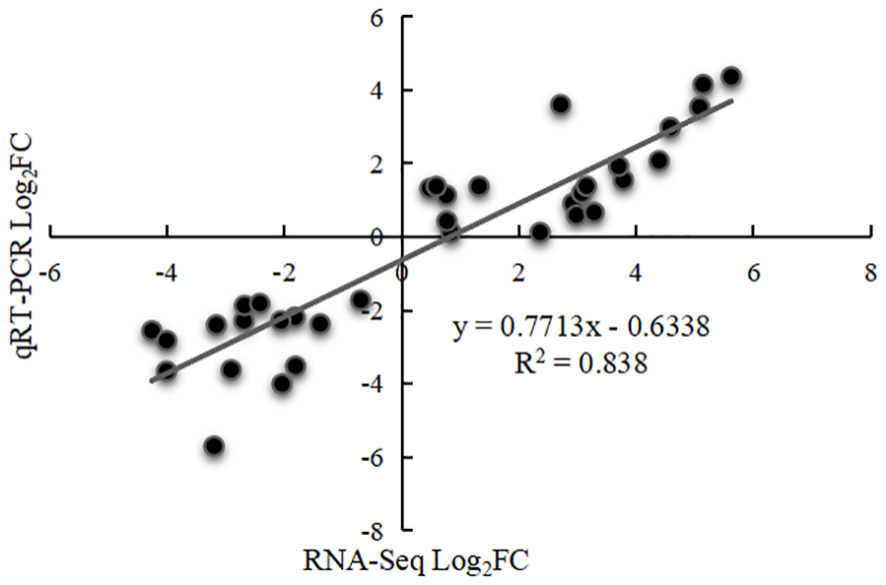
Correlation point map between RNA-Seq and qRT-PCR expression patterns.

To confirm the reliability of the transcriptome data, seven genes were selected for qRT-PCR verification. Comparison of transcriptome sequencing data and qRT-PCR data indicated very similar expression trends, with a Pearson correlation coefficient (R^2^) of 0.838 (**Figure 8**; **Supplementary Figure 1**), demonstrating good reliability for the RNA-seq data.

## 4 Discussion

The defenses initiated by plants after being attacked by herbivorous insects are induced defenses. The process of inducing insect resistance includes the activation of pest stress signals, transmission of internal pest signals, expression of defense compound-associated genes, and synthesis of defense substances, culminating in insect resistance (Stout and Duffey, 1996). Nutrients, secondary metabolites, and defense enzymes play a vital role in the physiological responses of plants to pest stress (War et al., 2013; Li et al., 2022b). Studies have shown that low levels of soluble sugars, amino acids, and other nutrients reduce the desirability for pests, and plant resistance is stronger (Cao et al., 2018). Insect damage induces plants to produce a large number of terpenoids, phenols, nitrogen-containing compounds, and other secondary metabolites, affecting insect feeding, growth, development, and reproduction (Divekar et al., 2022). Changes in the activities of defense-related enzymes occur during the production of secondary metabolites and other anthelmintic-related substances. Plant defense enzymes are upregulated in response to insect stress; they promote the synthesis of quinones, lignin, phytoalexins, and other insect resistance compounds in plants; hinder insect feeding; and maintain plant metabolic balance (Li et al., 2022a). Plant tissues usually contain a small amount of PIs. However, after being damaged by herbivorous insects, the damage site induces a large number of PIs to be rapidly transported throughput the plant, which blocks the protease activity in the intestine of herbivorous insects, thereby inhibiting pest population expansion and protecting the plant (Zhu-Salzman and Zeng, 2015). In previous studies, transcriptome analysis of plants in response to herbivorous insect feeding shown that DEGs were significantly enriched in hormone synthesis pathways such as biosynthesis of secondary metabolites (e.g., quinones and flavonoids), phenylalanine metabolism, POD activity, α-linolenic acid metabolism, and JA synthesis (Li et al., 2020).

Insects feeding on plants induce changes in primary and secondary metabolites, including sugars, amino acids, organic acids, flavonoids, phenols and tannins. In the present study, after feeding by *T. palmi*, the soluble sugars content in the leaves of daylily was significantly lower than that of the CK group at five timepoints. It may be that carbohydrates (mainly soluble sugars) synthesized in the aboveground part may not only meet the needs of plant growth, development, and defenses, but also be more distributed in the root system to ensure its growth activity, thereby improving the tolerance of the plant. Amino acid and fatty acid contents were higher than those in CK after 1 and 3 days of feeding by *T. palmi* and significantly lower than those in CK after 7 days. Amino acid and free fatty acid contents increased in the early stage of infestation, which may be a compensatory resilience of the plant to cope with pest infestation; however, when the infestation increased to a certain degree, the plant’s own nutrient supply was insufficient, and then there was a successive decrease in the contents of nutrients, finally lower than those in CK. It suggests that plants can become less attractive to pests through changes in nutrient levels in the body, and that nutrients can also be involved in defense responses to increase plant resistance to pests. Reduction of foliage nutritive quality after herbivory could be an adaptation of plants to insect attack, slowing down larval development and affecting negatively impacting insect fitness (Cornelissen and Stiling, 2006). Analysis of five cotton cultivars revealed that aphid and jassid infestation decreased each cultivar’s sugar and protein content (Amin et al., 2016). Notably, a previous study reported that the low sugar and protein content in tomato leaves is not conducive to the growth and development of *Helicoverpa armigera* (Bisht et al., 2022). Insect feeding induction is an important factor triggering the plant defense system. The elicitors in insect oral secretions enable plants to identify harmful signals, then initiate the defense system to induce resistance (Alves-Silva and Del-Claro, 2016). For example, through the catalysis of various defense enzymes such as POD, PAL, PPO, and PI, they induce the accumulation of various toxic secondary metabolites such as phenols, alkaloids, and terpenoids in plants, thereby directly or indirectly improving insect resistance (Appu et al., 2021). *Nilaparvata lugens* feeding increased the activities of POD, PAL, and PPO in rice plants, which not only reduced the damage induced by pest feeding, but also played an important role in the accumulation of toxic metabolites (Li et al., 2023b). *Pieris rapae* feeding causes damage to *Phaseolus vulgaris L.* leaves, which directly induces high expression of PI genes, and plants exhibit induced insect resistance (Xiang et al., 2018). In the present study, the activities of defense enzymes such as POD, PAL, PPO, and PI, and the contents of secondary metabolites such as tannins, flavonoids, and total phenols in leaves of daylily following feeding by *T. palmi* were significantly higher than those of CK. These findings are consistent with previous reports showing that thrips damage significantly increased the flavonoid, tannin, and lignin content in alfalfa leaves (Wu et al., 2021), and *H. armigera* feeding significantly increased the phenol content of pigeon pea (Kaur et al., 2014). In our previous study, PI activity was significantly increased in plants exposed to insect damage, resulting in the obstruction of insect digestion and slow growth, and the tannins, flavonoids, and total phenols content in daylily leaves were significantly higher in plants exposed to insect damage, which were not conducive to colonization by *T. palmi* (unpublished data).

After plants are stressed by insect feeding, defense signaling pathways are initiated, a series of physiological and biochemical reactions are induced, and the expression of defense genes is activated (Wu et al., 2010). In alfalfa damaged by thrips, pathways related to carbohydrate metabolism, lipid metabolism, MAPK signaling, hormone synthesis, and secondary metabolite synthesis are activated to initiate a defense response to thrips damage (Zhang et al., 2021). In the present study, the DEGs identified in daylily exposed to *T. palmi* infestation were mainly enriched in secondary metabolite synthesis, defense hormones signal transduction, defense enzymes synthesis, MAPK signaling pathway-plant, cell wall thickening, carbohydrate metabolism, photosynthesis, and other insect-resistant pathways. The transcription factors identified on the basis of DEGs were clustered into the MYB, bHLH, AP2/ERF, WRKY, bZIP, and NAC families. Among them, MYB, WRKY, bHLH, and AP2/ERF transcription factors were significantly upregulated after feeding by *T. palmi*, indicating that these four families of transcription factors play an important role in induced resistance to *T. palmi* defense in daylily. The aphid resistance-related transcription factors in alfalfa were consistent with the thrips resistance-associated transcription factors in daylily, but the MYB, NAC, and AP2/ERF families were dominant in alfalfa responses to aphids (Jacques et al., 2020). Furthermore, WGCNA and DEGs analysis demonstrated that MYB-like DNA-binding domain (TRINITY _ DN2391 _ c0 _ g1, TRINITY _ DN3285 _ c0 _ g1), zinc-finger of the FCS-type C2-C2 (TRINITY _ DN21050 _ c0 _ g2), and regulatory protein NPR1 (TRINITY _ DN13045 _ c0 _ g1, TRINITY _ DN855 _ c0 _ g2) are closely related to the synthesis of anti-stress compounds such as antioxidant enzymes, JA, SA and secondary metabolites. These results suggest that these genes play an important role in the defense responses of daylily to *T. palmi*.

In conclusion, the present findings elucidate the potential mechanism and hub genes of the resistance of daylily to *T. palmi*. The synergistic effects of nutrients, secondary metabolites, and defense enzymes increased the resistance of daylily to *T. palmi*. The mechanisms include reducing the nutrients available to *T. palmi*, catalyzing defense enzymes to produce secondary metabolites that are toxic to *T. palmi*, activating JA, SA, and other defense hormones signal transduction pathways, improving the resistance of daylily plants, and reducing the damage caused by *T. palmi*. The results of this study expand our the understanding of the mechanisms of insect resistance in daylily, and inform the development of effective strategies to control *T. palmi* by inducing exogenous factors to enhance insect resistance.

## Data availability statement

The data presented in the study are deposited in the NCBI repository, accession number PRJNA1094559.

## Author contributions

ZS: Conceptualization, Data curation, Formal analysis, Investigation, Methodology, Visualization, Writing – original draft. HS: Data curation, Visualization, Writing – original draft. ZC: Data curation, Methodology, Writing – original draft, Conceptualization, Formal analysis, Funding acquisition, Investigation, Project administration, Resources, Software, Supervision, Validation, Visualization. NM: Formal analysis, Visualization, Writing – original draft. YY: Data curation, Formal analysis, Writing – original draft. HL: Conceptualization, Methodology, Writing – original draft. JL: Conceptualization, Funding acquisition, Project administration, Resources, Supervision, Writing – review & editing.
