# Supplementary figures and images for: Physiological responses and transcriptome analysis of Hemerocallis citrina Baroni exposed to Thrips palmi feeding stress
Source: Front Plant Sci. 2024 May 14;15:1361276. doi: 10.3389/fpls.2024.1361276 (PMC11130412; doi:10.3389/fpls.2024.1361276)

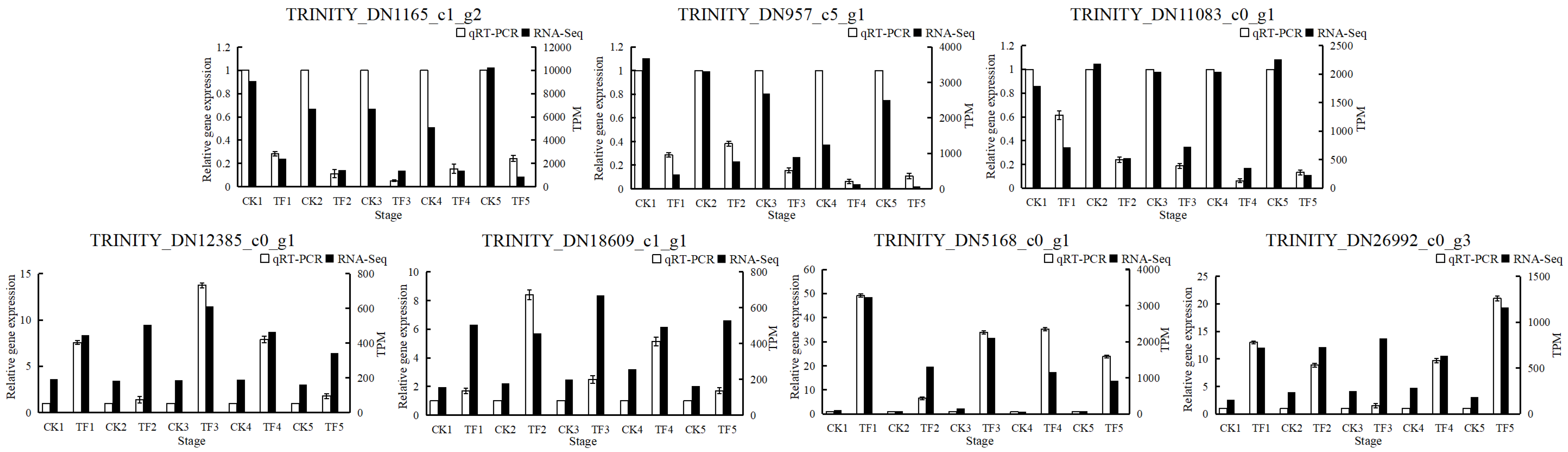

Supplement: Supplementary file 3 [file Image_1.tif]
